# Reconstruction of age distributions from differentially private census data

**DOI:** 10.1007/s11113-022-09734-2

**Published:** 2022-10-26

**Authors:** Sigurd Dyrting, Abraham Flaxman, Ethan Sharygin

**Affiliations:** 1 Northern Institute, Charles Darwin University, Darwin, NT 0909 Australia; 2 Institute for Health Metrics and Evaluation (IHME), University of Washington, 3980 15th Ave. NE, Seattle, WA 98195 USA; 3 Population Research Center, Portland State University, PO Box 751, Portland, OR 97207 USA

**Keywords:** Census, *Privacy*, Demography

## Abstract

The age distribution of a population is important for understanding the demand and provision of labor and services, and as a denominator for calculating key age-specific rates such as fertility and mortality. In the US, the most important source of information on age distributions is the decennial census, but a new disclosure avoidance system (DAS) based on differential privacy will inject noise into the data, potentially compromising its utility for small areas and minority populations. In this paper, we explore the question whether there are statistical methods that can be applied to noisy age distributions to enhance the research uses of census data without compromising privacy. We apply a non-parametric method for smoothing with naive or informative priors to age distributions from the 2010 Census via demonstration data which have had the US Census Bureau’s implementation of differential privacy applied. We find that smoothing age distributions can increase the fidelity of the demonstration data to previously published population counts by age. We discuss implications for uses of data from the 2020 US Census and potential consequences for the measurement of population dynamics, health, and disparities.

## Introduction

The age distribution of a population, indexed by location and sex, is an essential element of demographic analysis and government policy formation, used both directly to enumerate subpopulation size and indirectly in the calculation of key ratios such as age-specific rates of fertility and death, as well as associated summary measures such as the total fertility rate (TFR) and life expectancy at birth (Preston et al., 2001). Because the provision and demand for labor and services is strongly related to age, the age distribution is also the principal output of population forecasting models as well as an important input (Smith et al., 2013).

In the United States, the most important source of demographic data, including the population age distribution, is the Decennial Census of Population and Housing. As the US Census Bureau (USCB) depends on public trust for accurate and complete responses and is prohibited by law from facilitating reidentification of respondents (Title 13 U.S.C. §9, 2018), it has developed a set of procedures, collectively termed a disclosure avoidance system (DAS), designed to prevent the identification of individual census records from consolidated data (McKenna, 2018). In the face of increasingly sophisticated approaches for database reconstruction, the USCB has decided that for its flagship project, the publication of tabulations derived from the 2020 decennial census, its traditional statistical disclosure avoidance methods will be replaced by its TopDown Algorithm (TDA), a system based on differential privacy (DP) (Jarmin, 2019).

TDA takes as input the Census Edited File $T$, containing census responses along with corrections for errors and imputation of missing items. From $T$, TDA produces a randomized Privacy-Protected Microdata File $\tilde{T}$ which is used for all tabulation and data releases. The algorithm gives zero-Concentrated Differential Privacy (Bun & Steinke, 2016), a variant of differential privacy controlled by a privacy-loss parameter $\rho$ (abbreviated $\rho$-zCDP): the probability of inferring anything about a person will not increase by more than a known amount as a result of that person participating in the census. It should be emphasized that differential privacy is a statement about the worst-case *change* in probabilities rather than on their *level*, and that it is a privacy-loss accounting metric and not a specific randomization algorithm.^1^

There is a formal equivalence between a microdata file such as $T$ and a multi-dimensional histogram $H$, it being possible to represent the same data either as item values for each individual or as counts of individuals with each possible combination of item values. TDA achieves $\rho$-zCDP by converting $T$ to its equivalent histogram $H$, adding noise to histogram values with variance inversely proportional to a share of $\rho$ (as well as to additional queries for statistics that aggregate certain important marginals in the histogram), post-processing the result so that it satisfies certain constraints (for example, to be a valid histogram counts must be non-negative), and then converting the resulting histogram $\tilde{H}$ into its equivalent microdata file $\tilde{T}$. The algorithm is ‘top down’ because it implements the above process recursively, constructing noisy measurements with national information, partitioning the result by state then extending it with noisy counts by county, the process of partitioning and extending repeating down the geographical hierarchy and ending at the census block level (Abowd et al., 2019). The privacy-loss budget $\rho$ is adjustable within the range $0< \rho < \infty$, and the hierarchical form of the algorithm allows flexibility in apportioning the amount ‘spent’ at each geographical level (as well as for important marginals in the histogram).

Proponents of the USCB’s decision to update its disclosure avoidance system argue that the methods used for previous censuses were ad hoc, unquantified, opaque, and no longer provided the level of privacy protection required by law, and that TDA implements a solution that, through DP, can quantify the leakage of information in a way that is responsive to preferences on the risk of reidentification regardless of future events, within a formal and transparent framework (Garfinkel et al., 2018; Abowd, 2016, 2021). Opponents argue that the USCB has overstated both the feasibility and the utility to an adversary of a successful reconstruction attack on census data and has been unduly aggressive in implementing a system that incorrectly interprets Title 13 U.S.C. §9 (2018), and which will undermine the usefulness of the data for a wide range of purposes (Ruggles et al., 2018, 2019; Ruggles and Van Riper, 2021; National Academies of Sciences, Engineering, and Medicine, 2020; Santos-Lozada et al., 2020; Swanson et al., 2021). For example, others have found that demonstration data from the new disclosure avoidance system exhibit increasingly unrealistic shapes in age distributions as population size decreases (Nagle, 2020; Salvo, 2020; Spence, 2020). When these age distributions are used as denominators, it can lead to an increase in the dispersion of age-specific mortality rates (Hauer and Santos-Lozada, 2021).

While it is recognized that deployment of TDA will in many cases lead to age distributions with significant levels of noise, there are currently no proposed methods that explicitly treat them as noisy observations and which seek to infer the unobserved age structure given knowledge of the size of the noise, that is, to improve estimates of the age structure through smoothing. In this article, we ask: are there smoothing methods that can enhance the research uses of DP data without compromising privacy?^2^

In the next section, we adapt the P-TOPALS smoothing approach (Dyrting, 2020) to the problem of inferring an age distribution from noisy observations. In Sections 3 and 4, we apply the method to smooth county-level data from May 2020 and March 2022 demonstration data from the 2010 US Census and illustrate its effectiveness in improving estimates of the age distributions for small populations and reducing dispersion in estimates of age-specific rates and vital summary measures. In Section 5, we discuss implications for its practical implementation for Census 2020 tables.

## P-TOPALS for age distributions

Age smoothing is a particular type of smoothing of numeric vectors where the values correspond to the population at a given exact age or age interval. Population counts may exhibit high variability between ages, some of which is meaningful and some of which is noise or error. In the mid-20th century, researchers proposed methods to account for misreporting of age by census respondents while preserving accurate totals (Arriaga, 1968; United Nations, 1956). Smoothing methods have also been developed to accommodate age-structured processes such as fertility, mortality, and migration. Penalized B-splines (P-splines) have been a favored approach, requiring few parameters and with demonstrated usefulness in modeling and forecasting age-specific mortality rates (Eilers & Marx, 1996; Currie et al., 2004). However, P-splines cannot readily distinguish between roughness and meaningful inter-age variation to penalize only the former. The TOPALS approach (de Beer, 2011, 2012) uses an external standard age schedule to improve the fit of spline smoothing models to age schedules with a high degree of meaningful inter-age variation, but requires interactive adjustment of parameters. The P-TOPALS approach combines the advantages of the TOPALS and P-spline models (Dyrting, 2020).

An age distribution published in a census table consists of a set of population numbers

1 $$\begin{aligned} {\tilde{N}}=\left[ \begin{array}{c} {\tilde{N}}_1\\ \vdots \\ {\tilde{N}}_m\end{array}\right] \end{aligned}$$

over $$m$$ age intervals $$[a_i,a_i+n_i)$$ for $$i=1,\ldots ,m$$. Data are often tabulated for fixed intervals of one year ($$n_i=1$$), as in DHC table PCT1, or five years ($$n_i=5$$), as in DHC table P12 and race/ethnicity tables P12A-P12I, with the final age interval possibly open ($$n_m=\infty$$). The objective is to estimate the population at single years of age $$N_x$$, $$x=0,1,2,\ldots ,\omega$$ out to a maximum age $$\omega$$ under conditions where $${\tilde{N}}$$ contains noise added as part of a confidentialization process (Andersson et al., 2009; Thompson et al., 2013).

In P-TOPALS, the estimate is expressed relative to a prior age distribution ${\hat{N}}$

2 $$\begin{aligned} N_x = {\hat{N}}_x \, \exp \left( B_x\cdot \theta \right) , \end{aligned}$$

where $B_x$ is a row vector of B-splines (de Boor, 2001) evaluated at age *x*, $\theta$ is a column vector of weights to be determined. This form allows the user to include prior information about the age distribution into the estimation problem. This information might be in the form of specific knowledge of the components of population change (births, deaths, and net migration) which have been used to make a population estimate independent of the census data, or general views on the persistence of stationary features of the distribution due to the predominance of special populations with stable age distributions (Swanson and Tayman, 2012), or the propagation ‘up’ the age profile of non-stationary features associated with past major demographic events (Bouvier, 1980; West et al., 2014).

The weights $\theta$ are found by maximizing the penalized log likelihood function

3 $$\begin{aligned} \mathscr {L}(\theta ) = \mathscr {L}_N(\theta ) - \frac{\lambda }{2}\,\theta '\cdot D'\cdot D\cdot \theta \end{aligned}$$

where $D$ is the first order difference matrix (Eilers and Marx, 1996) and $\lambda$ is the roughness penalty. The first term on the right hand side of Equation 3 is the log likelihood of having the tabulated distribution ${\tilde{N}}$ conditional on the underlying true distribution being $N$. We assume for simplicity that the noise injected by TDA can be approximated by a normal distribution, in which case

4 $$\begin{aligned} \mathscr {L}_N(\theta )=-\iota '\cdot \frac{1}{2\sigma ^2}({\tilde{N}}-N)^2 , \end{aligned}$$

where $\sigma ^2$ can be age-dependent and $\sigma ^2\rightarrow 0$ as $\epsilon \rightarrow \infty$. Here, $\iota$ is a vector of ones and $N$ is the vector of $m$ smoothed numbers given in terms of $N_x$ by the sum

5 $$\begin{aligned} N_i = \sum _{a_i\le x<a_i+n_i} N_x,\quad i=1,\ldots ,m. \end{aligned}$$

The B-splines are defined on a relatively fine grid of knots and smoothing relative to the standard is achieved by the second term on the right of Equation 3 which penalizes first differences in the weights for adjacent splines.

Assuming $\mathscr {L}(\theta )$ is maximized at a stationary point we get the following nonlinear equation for $\theta$

6 $$\begin{aligned} G'(\theta )\cdot V\cdot ({\tilde{N}}-N)-\lambda D'\cdot D\cdot \theta =0, \end{aligned}$$

where

7 $$\begin{aligned} V= \mathrm {diag}(N/\sigma ^2), \end{aligned}$$

and $G(\theta )$ is the matrix of logarithmic derivatives

8 $$\begin{aligned} G(\theta ) = \frac{1}{N}\frac{\partial {N}}{\partial \theta }. \end{aligned}$$

Equation 6 can be solved by iterated linear regressions as shown in Dyrting (2020). The penalty $\lambda$ can be set manually or chosen using one of the criteria discussed in Dyrting (2020). In this article, we use the penalty that optimizes the Bayesian Information Criterion (Schwarz, 1978).

## Research design

USCB published demonstration data reflecting ongoing modifications to the DAS since October 2019. In May 2020, a Privacy-Protected Microdata File was published for population records, from which a number of the tables published from the 2010 Census release could be generated. The privacy-loss budget for person-level records in this release was $\epsilon$-differentially private with $\epsilon =4.0$ (Fontenot, 2019). In March 2022, a new release was published containing data in precompiled tables in the layouts proposed for the 2020 Census release, but generated with data from the 2010 Census with a more recent version of TDA applied, with $\rho$-zCDP with global privacy-loss budget of $\rho =5.885$, equivalent to $\epsilon =29.2$ under $\epsilon$ differential privacy (Hawes, 2022).^3^ From both releases, we produced or extracted tables corresponding to US county total resident population by sex and age (table PCT1) and by sex and age group for population by race/ethnicity (tables P12 and P12A-P12I). We selected counties because they vary widely in population size, from under 100 persons (Kalawao, HI) to 10 million (Los Angeles, CA).

We smoothed the DP demonstration data with two versions of P-TOPALS (PT). The default specification used a flat prior (PTF) with equal populations at each age. We also tested an estimates prior (PTE) derived from postcensal demographic estimates of the county population. The estimates are vintage 2009 county population and housing estimates from the Population Division of the US Census Bureau and contain single year of age and race detail derived from the 2000 census and cumulative births, deaths, and migration between 2000 and 2009 (National Center for Health Statistics, US Centers for Disease Control and Prevention, 2010). A naive assumption was made that 75 percent of the population would have experienced a birthday in a closed population during the nine months elapsed between July 1, 2009 (Estimates datum) and April 1, 2010 (Census Day). High rates of net migration in some counties mean that population churning or turnover would keep the age distribution constant. To increase the correspondence between the 2009 estimates to the expected age distributions in 2010, we defined a weight between 0 (stationary age distribution) and 0.75 (75 percent of the population advanced by 1 year) and linearly interpolated between 0 at age 40 and 0.75 at age 50, after which net migration is low in most counties.^4^ The B-spline knots were set equal to the age points of the respective distribution being smoothed. This ensures that as the privacy-loss budget increases, the smoothed distribution converges to the unsmoothed distribution ($\epsilon \rightarrow \infty$ or $\rho \rightarrow \infty$, $\sigma ^2\rightarrow 0$).

We do not have access to the 2010 Census Edited File, so instead we must compare the accuracy of the DP demonstration data and our smoothed data measured against published 2010 census data from the 100% Summary File 1 (SF) using root mean squared error (RMSE). Note that these data contain noise from the 2010 disclosure avoidance system, which will mean our values for RMSE will slightly overestimate the true values for both unsmoothed and smoothed data, and place a lower bound on the measured variance reduction of P-TOPALS. As a proxy for the variance parameter $\sigma ^2$ , we used the mean square difference between DP and SF age distributions. We apply the P-TOPALS method to DP demonstration data to determine whether reduction in RMSE is achieved for tables with age distributions by 1-year age groups by sex (Section 4.1) and age distributions by 5-year age groups by sex and race/ethnicity (Section 4.2). Finally, in Section 4.3, we evaluate the consequences for age-specific fertility and mortality rates derived from census population data, including associated summary measurements, the total fertility rate, and life expectancy.

Earlier studies have focused on demonstration data released in May 2020 which had a lower privacy-loss budget and therefore more noisy counts. One such study found that differences in total population between DP and SF were most evident among populations of 1,000 persons or less (Santos-Lozada et al., 2020). In their analysis, DP population denominators tended to reduce the estimated mortality rate for non-Hispanic Black and Hispanic population of any race. A subsequent study of COVID-19 mortality similarly showed that analysis would be impeded for studies using population denominators from DP for cohorts representing fewer than 2,500 persons, precluding analysis at the county level for many race and age groups (Hauer and Santos-Lozada, 2021). However, other research found that disparities by race in premature mortality (death before age 65) at the census tract level were insensitive to the choice of population denominators from the 2010 SF, ACS, or DP demonstration data (Krieger et al., 2021). The consequences of more accurate population counts for socioeconomic indicators and summary measures of population health could be significant. As a first step toward assessing these impacts, we calculate two county-level summary measures: the total fertility rate (TFR) and life expectancy at birth (${\dot{e}}_{o}$), to determine whether these measures are impacted by differential privacy. We calculate these by race and ethnicity for California counties: California is a large and diverse state with 58 counties ranging in population size from 1,000 (Alpine) to approximately 10 million (Los Angeles).

## Results

**Fig. 1:**
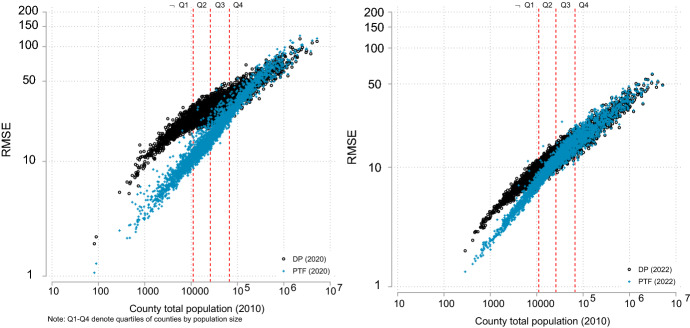
RMSE of 2010 population by age (PCT1): by county size

We first applied P-TOPALS to DHC single year of age tables PCT1, containing age and sex distributions for ages 0-100. We estimated an overall average RMSE of 31.1 for 3139 counties in the 2020-05 demonstration DP data prior to smoothing, and 13.8 in the 2022-03 data. The marked improvement in overall fidelity in the second release is attributable to algorithmic improvements in TDA as well as an increase in the privacy-loss budget from $\epsilon = 4.0$ to $\rho =5.885$ between releases. When we applied PT to smooth underlying age distributions, RMSE declined for 91 percent of counties in the 2020-05 release, including 99.5 percent of counties with fewer than 100,000 residents (Fig. 1). The 2022-03 release shows radical improvements over the prior release. The median US county population in 2010 was approximately 25,000 persons. In the 2022-03 release, PT reduced RMSE for 91 percent of counties with fewer than 25,000 residents and 99.4 percent of counties with fewer than 10,000 residents.

### Single years of age

Errors increase with county size, but not linearly: errors may be large relative to the population of small counties and trivial in large counties. The average cell count in the SF dataset of table PCT12 for a county with fewer than 10,000 residents is 26.5 persons. For a population that size, RMSE in the range of 10-25 indicates a coefficient of variation (CV=$\sigma /\mu$) of 38-90 percent, in excess of standards considered usable for census data (National Research Council, 2007; Environmental Systems Research Institute, 2011). In contrast, the CV in the 2022-03 release is typically below 10, with further improvements seen in the data with PT applied in both releases.

**Fig. 2:**
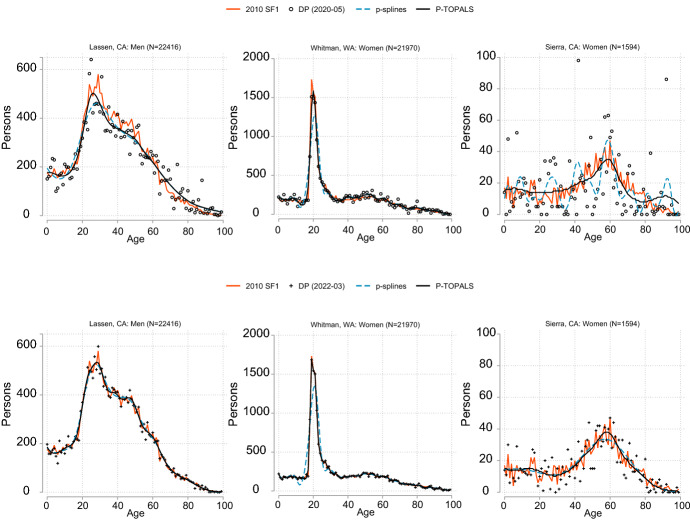
Raw and smoothed population by single year of age (PCT1): selected US counties

Single year of age data can be important for capturing dynamics about unique areas, including counties with a large share of population in a narrow band of ages. One such example is Whitman County, WA with an outsize proportion of its population that are students in dormitories (Fig. 2). The 2022-03 release of DP demonstration data shows that the current iteration of TDA copes well with this discontinuous age distribution. Lassen County, CA has a similar total population size, but is noisier. The noisiest age distribution is Sierra, CA, with just over 1,500 persons. The original 2010 SF1 data are also noisy (due in part to the 2010 DAS), but they retain characteristics of realistic age distributions and are less noisy than the figures from the current DAS for most counties. The PT smoothed data with flat prior agrees well with the published data for these counties and are more accurate than either the 2020-05 or 2022-03 releases for most counties with 25,000 and fewer persons. The results of smoothing with p-splines are shown for comparison. Demonstration data smoothed with p-splines showed poor agreement with published results for very noisy data (e.g., Sierra, CA) and where there are very large but meaningful discontinuities between single years of age (as in the case of Whitman, WA).

### Five-year age groups by race/ethnicity

Table P12 contains five-year age groups from 0 to 85 and older, with additional detail for several one or two-year age intervals around college ages (18-21) and ages 60-70. It is a widely used table series for small area population estimates because it contains valuable sex and age detail down to the census block level. Table P12 includes iterations by race/ethnicity (P12A-I), with additional race/ethnicity combinations proposed for tables in 2020. This makes the table a critical source of information on local area demographics.

Because they combine ages, P12 table series should have less deviation from the enumerated population counts than PCT1 tables. However, for tables P12A-I with race/ethnicity detail, the story may be different due to much smaller total population sizes in each table iteration. While only one county in the PCT1 series had a total population of less than 100, 29% of P12A-I tables contain fewer than 100 persons. Since we found in the previous section that RMSE declines non-linearly with total population size, this means that tables with race and ethnicity detail may be more difficult to report as enumerated for a given level of privacy.

**Table 1:** RMSE by race/ethnicity (P12, P12A-I): USA, 2010

		DP (2020-05)				
P12	Race	DP	PTF		PTE	
Table	or Ethnicity:	RMSE	RMSE	Shr<DP	RMSE	Shr<DP
B	Black	22.0	16.9	0.91	14.8	0.92
C	AIAN	10.4	6.3	0.98	5.4	0.98
D	Asian	12.0	8.8	0.94	7.8	0.94
E	NHPI	2.6	1.8	0.79	1.5	0.80
F	Other	16.1	10.8	0.98		0.98
G	Two or more	18.2	12.1	0.99	11.4	1.00
H	Hispanic	27.8	19.9	0.97	18.3	0.97
I	White (NH)	43.6	38.6	0.74	34.2	0.78
-	Total	66.9	61.2	0.76	52.8	0.87

		DP (2022-03)				
P12	Race	DP	PTF		PTE	
Table	or Ethnicity:	RMSE	RMSE	Shr<DP	RMSE	Shr<DP
B	Black	4.2	4.3	0.58	4.2	0.55
C	AIAN	3.3	2.8	0.91	2.9	0.81
D	Asian	3.0	2.8	0.77	2.9	0.70
E	NHPI	1.2	1.0	0.77	1.0	0.73
F	Other	3.6	3.3	0.77		0.77
G	Two or more	5.5	4.8	0.88	5.2	0.72
H	Hispanic	6.0	5.7	0.74	5.9	0.64
I	White (NH)	6.0	6.8	0.21	6.2	0.38
-	Total	8.9	9.7	0.27	9.0	0.42

		DP (2022-03)				
P12	Race	DP	PTF		PTE	
Table	or Ethnicity:	RMSE	RMSE	Shr<DP	RMSE	Shr<DP
B	Black	4.2	4.3	0.58	4.2	0.55
C	AIAN	3.3	2.8	0.91	2.9	0.81
D	Asian	3.0	2.8	0.77	2.9	0.70
E	NHPI	1.2	1.0	0.77	1.0	0.73
F	Other	3.6	3.3	0.77		0.77
G	Two or more	5.5	4.8	0.88	5.2	0.72
H	Hispanic	6.0	5.7	0.74	5.9	0.64
I	White (NH)	6.0	6.8	0.21	6.2	0.38
–	Total	8.9	9.7	0.27	9.0	0.42

Source: 2010 US Census Summary File 1; 2020–2005 and 2022–2003 DP Demonstration Data; authors’ calculations. Shr<DP is the share of counties for which P-TOPALS RMSE is lower than DP RMSE (out of a total of all US counties). AIAN refers to American Indian or Alaska Native and NHPI to Native Hawaiian or Pacific Islander. White NH refers to White, non-Hispanic. P12F is blank for PTE because no estimates prior was available for “Other” race alone

The results summarized in Table 1 for all U.S. counties exhibit improvements of between 8-40% in RMSE for P-TOPALS with flat prior and 20-50% for the estimates prior in the 2020-05 release. The 2022-03 DP release shows significant improvements in accuracy over the earlier release, with P-TOPALS smoothing resulting in smaller improvements on average. Even in the 2022-03 release, P-TOPALS with flat or estimates prior outperformed DP in the majority of counties for all tables except P12I (White alone, not Hispanic). For table P12I, the average county population size was over 60,000 persons, and although P-TOPALS had higher RMSE, the CV remained small, implying only minor differences between DP and P-TOPALS relative to population size.

### Key rates and summary measures

Total fertility rate (TFR) is an aggregation of age-specific birth rates, representing the live births per woman to a hypothetical cohort who experienced all period age-specific birth rates. Fertility rates inform public policy discussions and have resulted in changes in taxation and labor laws, among other things.

Table 2 shows the TFR estimated from the average births during 2009-11 for counties in California (N=58). Values of TFR are calculated for each of 7 single values of race or ethnicity in each county.

**Table 2:** TFR by race/ethnicity: California, 2010

			DP (2020-05)					
P12	Race	SF:	DP		PTF		PTE	
Table	or Ethnicity:	TFR	TFR	RMSE	TFR	RMSE	TFR	RMSE
B	Black	1.80	1.81	0.03	1.80	0.02	1.80	0.02
C	AIAN	1.02	1.16	0.20	1.02	0.07	1.03	0.08
D	Asian	1.64	1.64	0.01	1.64	0.01	1.64	0.01
E	NHPI	2.06	2.15	0.24	2.11	0.19	2.16	0.16
G	Two or more	1.13	1.18	0.08	1.12	0.04	1.14	0.04
H	Hispanic	2.30	2.31	0.02	2.30	0.01	2.31	0.01
I	White (NH)	1.67	1.67	0.01	1.67	0.01	1.67	0.01
-	Total	1.96	1.96	0.01	1.96	0.01	1.96	0.01

			DP (2022-03)					
P12	Race	SF:	DP		PTF		PTE	
Table	or Ethnicity:	TFR	TFR	RMSE	TFR	RMSE	TFR	RMSE
B	Black	1.80	1.80	0.01	1.80	0.01	1.80	0.01
C	AIAN	1.02	1.03	0.02	1.02	0.02	1.02	0.02
D	Asian	1.64	1.64	0.00	1.64	0.00	1.64	0.00
E	NHPI	2.06	2.06	0.05	2.06	0.04	2.08	0.05
G	Two or more	1.13	1.13	0.01	1.12	0.01	1.13	0.01
H	Hispanic	2.30	2.30	0.00	2.30	0.00	2.30	0.00
I	White (NH)	1.67	1.67	0.00	1.67	0.00	1.67	0.00
-	Total	1.96	1.96	0.00	1.96	0.00	1.96	0.00

Source: 2010 US Census Summary File 1; 2020–2005 and 2022–2003 DP Demonstration Data; CA Dept of Public Health; authors’ calculations. Population weighted estimates. To improve the reliability of TFR estimates for small populations, average births per age group during the period 2009–2011 were used instead of 2010 alone. Births to women aged <15 or >49 were recoded to 15 or 49, respectively. Table F was omitted because no data were available for births to mothers of “Other” race alone

**Fig. 3:**
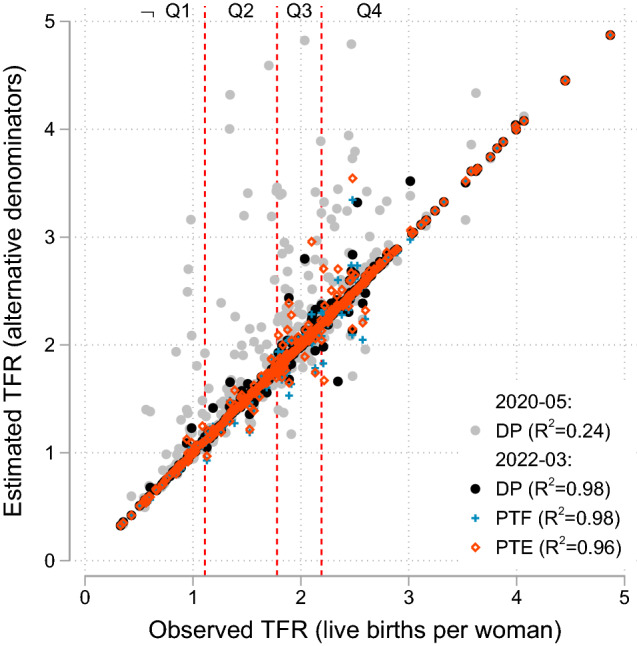
Dispersion of estimates by TFR level: California counties, 2010

TFRs calculated from P-TOPALS population denominators were more accurate on average than those calculated using DP demonstration data. This is especially true for the 2020-05 release, where the TFRs calculated using DP denominators exhibited upward bias, especially evident in unweighted TFR estimates (Fig 3).^5^ In the 2022-03 release, the DP data showed greatly improved accuracy over the 2020-05 release: the correlation between the TFRs derived from published 2010 Census data and the DP demonstration data releases increased from 0.24 to 0.98 between the 2020-05 and 2022-03 releases, and the improvement from P-TOPALS is less noticeable in the more recent release.

Life expectancy is another important summary measure widely used to inform discussions on population health and public finance. Population denominators used in mortality measurement could also have an impact on the calculation of period life expectancy. In recognition of the importance of a long and healthy life, life expectancy is, together with education and economic output, used to calculate the Human Development Index, a measure of the freedom and opportunity of populations (United Nations Development Programme, 2020).

P-TOPALS variants improve upon DP for accurate measurement of life expectancy at birth (${\dot{e}}_{o}$) by race/ethnicity (Table 3). The improvement made by P-TOPALS on the measurement of life expectancy is less compared to TFR due to minimum population size criteria to calculate a life table (15,000 person-years and at least 700 deaths). For California’s 58 counties, a total of N=116 life expectancy at birth estimates are possible for each P12 table iteration, but only 87 tables meet the publication criteria. Nonetheless, improvements were evident in the 2020-05 release for tables B (Black alone) and H (Hispanic, any race). The flat and estimates priors performed equally well, with a slight edge to the flat prior, and no significant bias. The 2022-03 release eliminated any significant error in life expectancy calculations in the demonstration data.

**Table 3:** Life Expectancy by race/ethnicity: California counties, 2010

			DP (2020-05)					
P12	Race	SF:	DP		PTF		PTE	
Table	or Ethnicity:	${\dot{e}}_{o}$	${\dot{e}}_{o}$	RMSE	${\dot{e}}_{o}$	RMSE	${\dot{e}}_{o}$	RMSE
B (17)	Black	74.68	74.33	0.33	74.40	0.27	74.34	0.31
D (19)	Asian	85.27	84.97	0.28	84.99	0.27	84.99	0.27
H (34)	Hispanic	82.95	82.46	0.51	82.65	0.37	82.80	0.45
I (82)	White (NH)	78.70	78.69	0.05	78.69	0.05	78.69	0.05
- (87)	Total	80.67	80.61	0.16	80.63	0.16	80.63	0.14

			DP (2022-03)					
P12	Race	SF:	DP		PTF		PTE	
Table	or Ethnicity:	${\dot{e}}_{o}$	${\dot{e}}_{o}$	RMSE	${\dot{e}}_{o}$	RMSE	${\dot{e}}_{o}$	RMSE
B (17)	Black	74.68	74.69	0.03	74.69	0.03	74.69	0.03
D (19)	Asian	85.27	85.25	0.03	85.25	0.03	85.25	0.03
H (34)	Hispanic	82.95	82.94	0.04	82.94	0.04	82.94	0.04
I (82)	White (NH)	78.70	78.70	0.01	78.70	0.01	78.70	0.01
- (87)	Total	80.67	80.67	0.01	80.67	0.01	80.67	0.01

Source: 2010 US Census SF1; 2020–2005 and 2022–2003 DP Demonstration Data; CA Dept of Public Health; authors’ calculations. Population weighted estimates. Life expectancy calculated at birth for counties with 700 or more deaths during 2009-11 and 15,000 person-years lived in 2010. Number of valid measurements indicated in parentheses

**Fig. 4:**
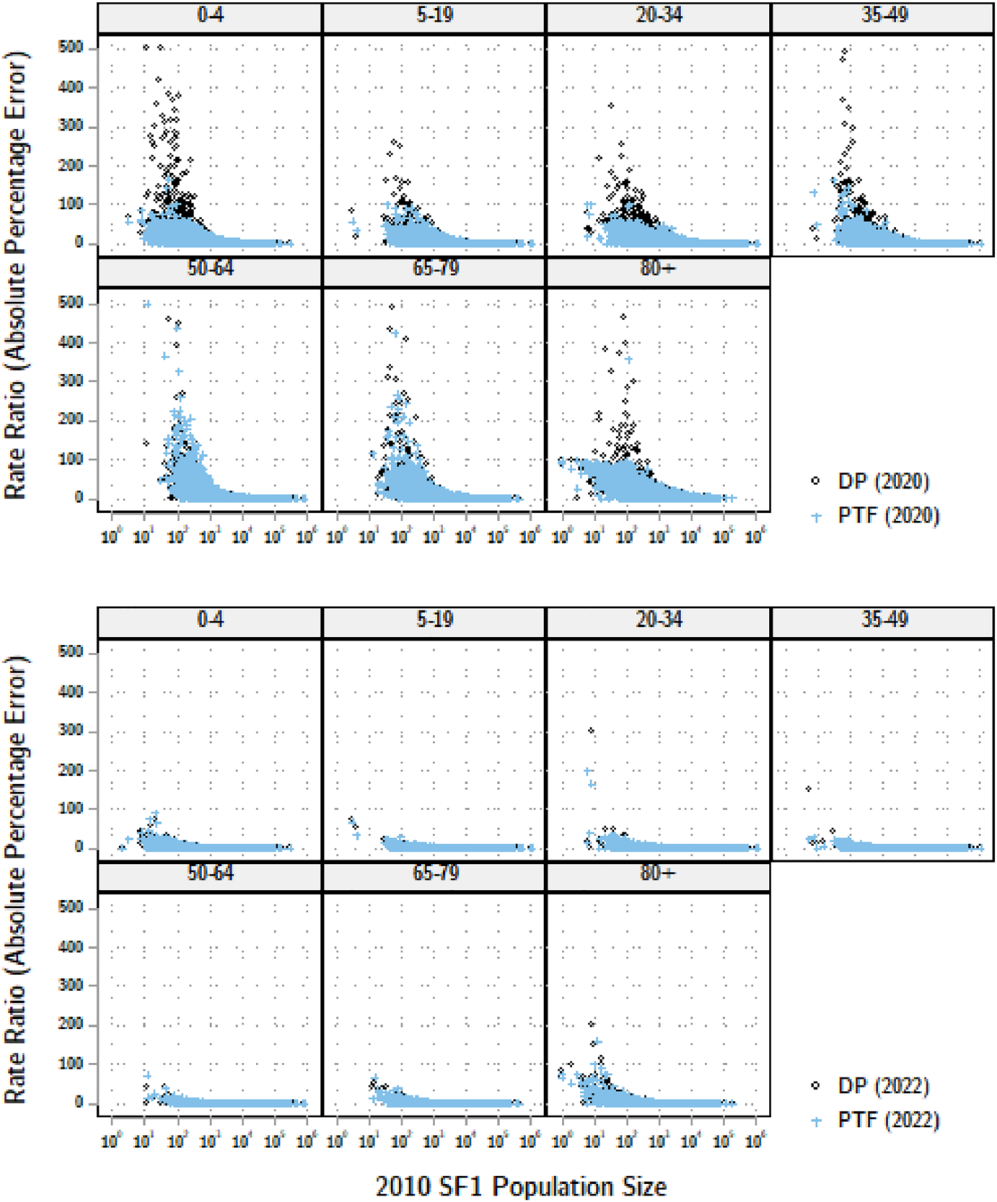
Errors in county age-specific rates: USA, 2010

There are many small counties where life expectancy cannot be reliably estimated using classical methods. Also, large counties may still have small populations when stratified by age and sex. We generalize our analysis to any age-specific rates by calculating the ratio $|{SF/DP}-1|$ or $|{SF/PTF}-1|$ for the absolute percent errors (APE) from the DP demonstration data or from P-TOPALS smoothed data, respectively, for age groups 0-4, 5-19, 20-34, 35-49, 50-64, 65-79, and 80 and over (Fig. 4).^6^

Accuracy increases remarkably between the two releases, with an immense reduction in the number of estimates with APE in excess of 100 percent. The P-TOPALS estimates are far less likely to be over 100 percent, and are generally more accurate for rates calculated for populations of less than 1,000 persons. This has important ramifications for estimates of racial disparities: nearly two-thirds of county total populations by race in the 2020 US Census were enumerated at fewer than 1,000 persons, and a majority of counties for Asian, Black, American Indian or Alaska Native, and Native Hawaiian or Pacific Islanders, even before stratifying by age and sex (Table 4).

**Table 4:** Counties by population size by race/ethnicity: USA, 2020

Race or ethnicity:	Percentile:			N $\le$ 1,000 persons	
	25th	Median	75th	Count	Share
Hispanic (any race)	358	1274	6515	1458	0.453
Non-Hispanic:					
Asian	32	126	770	2513	0.780
Black	72	682	5091	1762	0.547
AIAN	31	92	362	2812	0.873
NHPI	1	7	32	3155	0.980
White	7192	18709	50316	154	0.048

Source: 2020 Census P.L. 94-171 Redistricting Data File. Two or more races or other race not shown

## Discussion and conclusion

P-TOPALS, a novel smoothing method, requires only a roughness parameter, information on the variance of DP noise added, and a standard age distribution. A differentially private dataset of population by single year of age from the 2010 Census (DHC table PCT1) initially had a CV from DP noise in excess of 35 percent for counties with fewer than 10,000 residents (over one quarter of US counties in 2010). Applying P-TOPALS smoothing to this demonstration data release from May 2020 reduced the CV to reliable levels, without reducing the reliability of population estimates for other counties. Using a naive age prior, P-TOPALS showed greater agreement with published summary files than DP data for 91% of county age distributions, and 99.5% of counties with fewer than 100,000 persons.

New demonstration data released in March 2022 release shows that changes to the differential privacy implementation and large increases in the privacy-loss budget translate to very significant improvements in accuracy, but accuracy of age distributions for small populations remains problematic. P-TOPALS smoothing improved the fidelity of age distributions in 91% counties below the median population size of approximately 25,000 persons, and for 99.4% of counties with fewer than 10,000 residents.

Smoothing via P-TOPALS also increased the fidelity of P12 tables for population by race/ethnicity with age and sex detail. These tables are widely used for purposes as varied as stratified sample design (ensuring surveys are representative of the population at large) and measurement of health and economic disparities. Information on the age structure and race of the population is incorporated into measures such as the CDC Social Vulnerability Index (SVI) that may be used to prioritize billions of dollars of future public investments. Summary measures such as life expectancy and total fertility rate, as well as age-specific rates, show great improvement in the 2022-03 demonstration data. Still, we found that rates calculated using DP population denominators smoothed with P-TOPALS were more accurate than untreated DP data for populations under 1,000 persons.

While the production setting of the privacy-loss budget may be higher than the value used in the demonstration products and the USCB continues to improve TDA, no indications of an increase have been made so far. In either case, we believe that smoothing with P-TOPALS will still be valuable for geographies at the county level and below. The primary improvements to TDA so far consist of a change from Laplacian to Gaussian distributed noise (which reduced the occurrence of large outliers, thus reducing post-processing biases), and mitigating how noise propagates into geographies that are not part of TDA’s hierarchy. However, it is likely that DP noise will remain an issue for many counties and most sub-county geographies and minority populations. Even in the latter release, counties that benefit from P-TOPALS smoothing have populations spanning two orders of magnitude (Figure 1)

Our results support the case that smoothing should be applied to the age distributions of the 2020 census DHC tables PCT1 and P12 prior to decision-making. To realize the greatest benefit, consideration should be given to applying P-TOPALS smoothing prior to other post-processing steps so that the variance of DP noise can be more accurately modeled from DP parameters and results can be centrally disseminated rather than applied at individual users’ discretion. In addition, further work could lead to more informative priors for fitting P-TOPALS.

Our work also highlights the need for USCB to release additional metrics for the 2020 Census data (for example, the variance of the added noise, $\sigma ^2$). To facilitate research into improved methods for statistical analysis, the USCB might consider publishing noisy measurements file without other post-processing steps applied, and pre-DP/post-DP reference tables with the 2020 privacy settings using historical or synthetic microdata files which could be used by researchers for validation purposes.

## Footnotes

1 For a more thorough but accessible discussion of differential privacy, see (Bowen, 2021; Woods et al. 2018).

2 A variant of this question was first posed by Nissenbaum (National Academies of Sciences, Engineering, and Medicine, 2020)[pp. 94–95].

3 Combining the privacy-loss budgets from the P.L. 94-171 Redistricting Data and the DHC Person Data, and excluding the privacy-loss budget of on the DHC Housing Unit Data.

4 The 2010 Evaluation Estimates are postcensal estimates produced with a 4/1/2010 datum; however, they are not available with single year of age detail.

5 A 45$^{\circ }$ angle in Fig 3 indicates perfect correlation between the estimated and empirical TFR; points above the line indicate overestimates.

6 Hauer and Santos-Lozada (2021) presented hypothetical errors if COVID-19 mortality rates were analyzed using DP denominators, using the ratio $|ASDR^{DP}/ASDR^{SF} -1|= \left|\frac{D/DP}{D/SF} -1\right|$ where *D* is the number of deaths, which simplifies to $|SF/DP -1|$. Fig. 4 excludes ratios over 500 percent errors, affecting fewer than 40 estimates out of 44,000.
